# *HLA-DRB1* allele distribution in Chilean population: insights into rheumatoid arthritis susceptibility and protection

**DOI:** 10.3389/fimmu.2025.1594723

**Published:** 2025-05-13

**Authors:** Diego Catalán, Lilian Soto, Óscar Neira, María C. Cuéllar-Gutiérrez, Roberto Díaz-Peña, Octavio Aravena, Eduard Palou, Montserrat Carrascal, Juan C. Aguillón, Jaxaira Maggi

**Affiliations:** ^1^ Immune Regulation and Tolerance Research Group (IRT Group), Programa Disciplinario de Inmunología, Instituto de Ciencias Biomédicas, Facultad de Medicina, Universidad de Chile, Santiago, Chile; ^2^ Sección Reumatología, Departamento de Medicina Interna, Hospital Clínico de la Universidad de Chile, Santiago, Chile; ^3^ Hospital del Salvador, Universidad de Chile, Santiago, Chile; ^4^ Facultad de Ciencias de la Salud, Universidad Autónoma de Chile, Talca, Chile; ^5^ Immunogenetics Lab, Fundación Pública Galega de Medicina Xenómica, Servizo Galego de Saúde (SERGAS), Grupo de MedicinaXenómica-Universidade de Santiago de Compostela (USC), Instituto de investigación Sanitaria de Santiago de Compostela, Santiago de Compostela, Spain; ^6^ Servicio de Inmunología, Hospital Clínic de Barcelona, Barcelona, Spain; ^7^ Biological and Environmental Proteomics Group, Institute of Biomedical Research of Barcelona, Spanish National Research Council (IIBB-CSIC/IDIBAPS), Barcelona, Spain

**Keywords:** rheumatoid arthritis, HLA-DRB1 alleles, shared epitope, anti-CCP antibodies, Chilean cohort

## Abstract

**Introduction:**

Rheumatoid arthritis (RA) is an autoimmune disease influenced by genetic factors, particularly *HLA-DRB1* alleles. The objective of this study was to characterize the distribution of *HLA-DRB1* alleles in Chilean RA patients and healthy controls (HC) and evaluate associations with susceptibility or protection, autoantibody seropositivity, and disease activity.

**Methods:**

We genotyped 367 RA patients and 623 HC for *HLA-DRB1* using PCR-SSO. Then, we examined allele frequencies and distribution, including known RA risk alleles of the “Shared Epitope” (SE) of HLA-DRB1 and protective (PR) alleles, using the Chi-square or Fisher’s exact tests. Odds ratios with 95% confidence intervals were calculated to measure the degree of association, and unpaired T-tests were used to compare continuous variables.

**Results:**

The most frequent SE alleles among RA patients were *04:01 (16.1%), *04:04 (13.9%), and *14:02 (11.7%). SE alleles *04:01, *04:04, *04:05, *04:08, and *10:01, along with non-SE alleles *09:01 and *15:02, were associated with RA susceptibility. In addition, allele *14:02 showed an association with the presence of anti-cyclic citrullinated peptides (anti-CCP) antibodies. Meanwhile, PR alleles *11:01 (14.8%) and *16:02 (9.8%) were observed most frequently in HC and RA patients, respectively. PR alleles *11:01, *11:04, and *13:01, as well as the non-PR alleles *15:01, *04:07, *03:01, *07:01, and *08:02, were associated with protection from RA, and showed no significant associations with autoantibody seropositivity.

**Discussion:**

This study provides a comprehensive overview of *HLA-DRB1* allele distribution in the Chilean population, identifying both well-known and novel allele associations with RA susceptibility, protection, and disease activity.

## 1 Introduction

Rheumatoid arthritis (RA) is a systemic autoimmune disease, primarily targeting synovial joints, that affects between 0.1% and 2% of the population worldwide (1), and around 0.6% of the population living in Chile (2). The risk of developing RA, as well as the clinical presentation of the disease, is strongly influenced by genetic factors, which show substantial heterogeneity among different ethnicities (3).

The best-characterized gene associated with RA susceptibility and severity is *HLA-DRB1*, which encodes the β chain of the class II antigen presenting molecule HLA-DR (4, 5). A group of *HLA-DRB1* alleles encoding a conserved amino acid sequence at positions 70-74 (QKRAA, QRRAA, or RRRAA) of the third hypervariable region (HVR3) of the HLA-DR β chain, known as “Shared Epitope” (SE), has been widely associated with RA risk (6). Furthermore, this association is limited to a subset of patients that harbor RA-specific anti-citrullinated protein antibodies (ACPA), accounting for the most severe RA cases (7). It has been proposed that SE-containing HLA-DR molecules can accommodate citrulline but not arginine at their positively charged P4 pocket in HVR3, leading to presentation of citrullinated peptides to CD4+ T cells and ultimately to ACPA responses (7). In extensive studies involving populations with European ancestry, the SE alleles found to be most significantly associated with ACPA-positive RA risk follow an effect size hierarchy and are part of the allelic groups HLA-DRB1*04 (*04:01, *04:04, *04:08), HLA-DRB1*10 (*10:01), and HLA-DRB1*01 (*01:01) (8, 9). Likewise, ACPA-positive RA among East Asian populations is associated with HLA-DRB1*04 SE alleles, showing a prominent effect of the *04:05 allele, with HLA-DRB1*10:01, and to a lesser degree with HLA-DRB1*01:01 (10–12). In addition, among East Asian populations, an association of the non-SE allele HLA-DRB1*09:01 with ACPA-positive RA has also been described (12, 13). Meanwhile, the allele *14:02 has been associated with RA in American populations, including Mexicans (14), Peruvians (15), and Native North Americans (16).

It is equally important to recognize alleles that confer protective effects in RA. In this sense, the best-studied *HLA-DRB1* protective (PR) alleles for RA are known to encode an aspartic acid at position 70 (D alleles), leading to the sequences DRRAA and DERAA at positions 70-74 (17–19). The PR alleles include members of the HLA-DR*13 group (*13:01, *13:02, and *13:04) and HLA-DR*11 group (*11:01, *11:02, and *11:04), as well as the alleles *01:03, *04:02 and *12:01, which are associated with a low risk of developing RA and a milder disease (20, 21). It has been suggested that this protective effect could reflect a greater efficiency of these alleles to bind the CLIP peptide from the invariant chain (Ii, CD74), which would prevent the premature occupation of the antigen-binding groove by endogenous peptides (22).

Few reports have examined the distribution of *HLA-DRB1* alleles in the Chilean population. In these studies, the most frequent alleles were found to be DRB1*03:01, *07:01 (23, 24), *04:03, *08:02, *14:02, and *16:02 (24). Meanwhile, studies involving Chilean RA patients have shown that the most frequent SE alleles among this group are DRB1*04:01 (25), *01:01, and *04:04 (25, 26). Additionally, the SE alleles *04:01, *10:01 (25), *04:04, and *04:08 (26) have been associated with RA in this population. Of note the non-SE *HLA-DR9* alleles have also been found to be significantly associated with RA in Chilean individuals (25), particularly among rheumatoid factor (RF) seropositive patients (27). Regarding potential PR alleles, the non-D alleles *07:01 and *08:02 were found to confer protection against ACPA-positive RA in the Chilean population (25).

In this work, we explored the distribution of *HLA-DRB1* alleles in cohorts of Chilean RA patients and healthy controls (HC) and their association with susceptibility or resistance to develop the disease, as well as their association with autoantibodies seropositivity and disease activity. As Chile has a diverse ethnical composition, influenced by Native-American, African, and European ancestries, this study was limited to the central Metropolitan and Maule regions, characterized by the highest European and lowest Native-American ancestry proportions (28).

## 2 Methods

### 2.1 Patients and controls

To conduct this cross-sectional study, blood samples from RA patients and HC were collected between 2014 and 2024. In total, 367 RA patients were recruited: 129 from *Hospital Clínico de la Universidad de Chile* (HCUCH) and *Hospital del Salvador* (HDS) in the Metropolitan region (RA-cohort-I), and 238 from *Hospital de Talca* (HT) and the Rheumatology Unit of the Health Network at *Pontificia Universidad Católica de Chile* (PUCH) in the Maule region (RA-cohort-II). All patients fulfilled the 2010 ACR/EULAR criteria for RA diagnosis (29). Patients receiving biological or targeted synthetic disease-modifying anti-rheumatic drugs (DMARDs) for the last six months before recruitment were excluded from this study. Clinical and laboratory data were collected, including gender, age, and seropositivity for anti-cyclic citrullinated peptides (CCP) antibodies (the whole RA-cohort-II was seropositive for anti-CCP) and RF (only for RA-cohort-I). The Disease Activity Score based on 28 joint counts (DAS28) was assessed in RA-cohort-I at the time of sampling, where a DAS28 value below 2.6 indicates remission (30), 2.6-3.2 corresponds to low disease activity, and values above 5.1 indicate high disease activity. Additionally, blood samples were obtained from 623 HC recruited from hospital staff and voluntary donors who self-reported no history of autoimmune disorders or chronic inflammatory diseases. Medical anamneses and simple physical examinations were conducted. Individuals with suspected symptoms or self-reported for autoimmune or chronic inflammatory diseases were excluded. Of these 623 HC, 213 were from the HCUCH and the *Biobanco de Tejidos y Fluidos de la Universidad de Chile* (BTUCH) in Metropolitan region (HC-cohort-I), and 410 from *La casa del donante* (LCD) in Talca, Maule region (HC-cohort-II). All subjects signed an informed consent form in accordance with the Declaration of Helsinki, and the study protocol was approved by the Ethics Committees from all institutions involved (Approval N° 077-2013, N° 24–2028 and N° 27–2022 for HCUCH; N° 121–2018 and N° 63–2022 for HDS; N° 42–2021 for BTUCH; and N° 04/2014 for HT, PUCH and LCD).

### 2.2 DNA isolation

DNA from RA-cohort-I and HC-cohort-I was isolated from whole blood samples according to the salting-out procedure (31). Briefly, blood was mixed with lysis buffer (10 mM Tris, 10 mM KCl, 10 mM MgCl_2_, and 2 mM EDTA, pH 7.6, in the presence of 2.5% Triton X-100). After centrifugation, the pellet was washed and incubated at 55 °C in a buffer containing 10 mM Tris, 10 mM KCl, 10 mM MgCl_2_, 0.4 M NaCl, and 2 mM EDTA (pH 7.6), along with 10% SDS and proteinase K (10 µg/µL). After 10 min, 6 M NaCl was added to precipitate proteins, and the DNA-containing supernatant was transferred to a fresh tube for precipitation using ethanol. Genomic DNA from RA-cohort-II and HC-cohort-II was extracted using the GeneJET Genomic DNA purification kit #K0722 (ThermoFischer Scientific, Waltham, MA, USA), following the manufacturer’s protocols. The concentration of the purified DNA was measured using a BioTek Synergy spectrophotometer (Agilent, Santa Clara, CA, USA). Yields above 100 ng/µL with an A260/A280 ratio greater than 1.8 were used for further analysis. DNA integrity was also confirmed by running an aliquot on a 1% w/v agarose gel.

### 2.3 PCR-SSO analysis

*HLA-DRB1* gene typing was performed on DNA samples by PCR-SSO (Sequence Specific Oligonucleotides) reverse technique, using the Lifecodes HLA-DRB1 SSO Typing Kit and following the manufacturer’s instructions (Immucor-Werfen, Waukesha, WI, USA). Briefly, *HLA-DRB1* exon 2 was amplified and subsequently hybridized with DNA probes covering the main polymorphic positions of the gene. A Luminex FLEXMAP 3D instrument and the xPonent software (ThermoFischer Scientific) were used for data acquisition. HLA typing analysis was achieved using the MATCH IT! DNA version 1.3 software suite (Immucor-Werfen). Intermediate resolution HLA typing results obtained by this technique provide an approximation of the most probable alleles (though not offering definitive high-resolution confirmation) in accordance with the classification of an allele as common (frequency ≥1 in 10,000) (32).

### 2.4 Statistical analysis

Allele frequencies were compared between groups using the Chi-square or Fisher’s exact tests. Odds ratios (ORs) were calculated with 95% confidence intervals (CI) in order to study associations between the presence of the disease or seropositivity in patients, with the presence of a specific *HLA-DRB1* allele or a group of *HLA-DRB1* alleles. The normality of continuous variables was evaluated by the Kolmogorov-Smirnov test. Comparisons between continuous variables were performed using the unpaired T-test. A p-value less than 0.05 was considered statistically significant. IBM SPSS Statistics version 29.0.2.0 (New York, NY, USA) was used for statistical analysis. GraphPad Prism version 8.3.0 (Boston, MA, USA) was used for the preparation of graphs.

## 3 Results

### 3.1 Characteristics of the study populations

The most relevant characteristics of the RA and HC cohorts included in the study are shown in **Table 1**. Participants were predominantly female (84% and 74% of RA patients and HC, respectively), with ages of 49 ± 12 years for patients and between 39 ± 9 years for controls. While all patients from RA-cohort-II were seropositive for anti-CCP, most patients from RA-cohort-I were seropositive for anti-CCP (54%) and RF (68%) and exhibited an active disease (70% had a DAS28 > 2.6).

**Table 1:** Main characteristics of RA patients and HC.

	RA patients (n=367)	HC (n=623)
Age, mean ± SD years	49 ± 12	39 ± 9
Gender, % of females/males	84/16	74/26
DAS28, mean ± SD *	4 ± 2	–
RF, % of positive/negative/n.a.*	68/13/19	–
Anti-CCP % of positive/negative/n.a.**	54/12/34	–

*Data available only from RA-cohort-I; **Data shown only for RA-cohort-I, as 100% of RA-cohort-II patients tested positive for anti-CCP. RA, rheumatoid arthritis; HC, healthy controls; SD, standard deviation; DAS28, disease activity score in 28 joints; RF, rheumatoid factor; n.a., not available; anti-CCP, anti-cyclic citrullinated peptides antibodies.

### 3.2 Frequencies of SE alleles in Chilean RA patients and HC

Subjects presenting at least one of the following alleles were considered positive for the SE: HLA-DRB1*01:01, *01:02, *04:01, *04:04, *04:05, *04:08, *04:10, *10:01, *14:02, *14:06, and *14:13 (21). When it was not possible to discriminate between *04:04, *04:05, *04:08, and *04:10 alleles, carriers were nonetheless considered positive for the SE.

**Figure 1:**
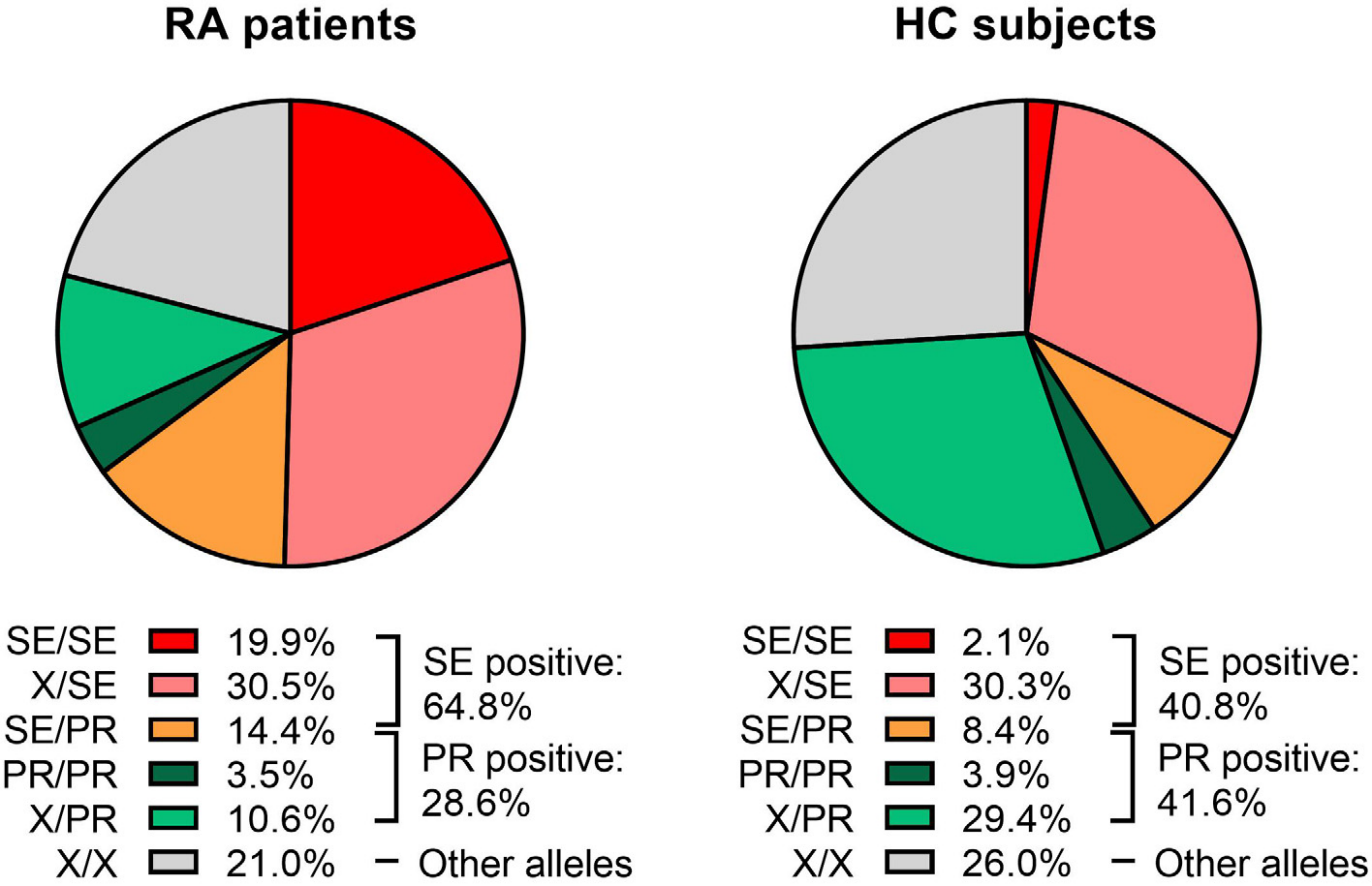
Distribution of *HLA-DRB1* genotypes in rheumatoid arthritis (RA) patients and healthy controls (HC). Each pie chart is divided according to the presence or absence of “Shared Epitope” (SE) and Protective (PR) alleles. The following genotype combinations are depicted: SE/SE (red), X/SE (pink), SE/PR (orange), PR/PR (dark green), X/PR (light green), and X/X (gray), where “X” denotes an allele that is neither SE nor PR. Percentages indicate the proportion of subjects within each category. RA patients (left pie chart) exhibited higher frequencies of SE-containing genotypes than HC (right pie chart). The overall percentages of SE-positive and PR-positive individuals are provided to the right of each pie chart.

A total of 64.8% of RA patients carried at least one copy of the SE, versus 40.8% of HC (OR=2.7; 95% CI=2.1-3.5; p<0.001). When SE allele dosage was analyzed, 44.9% of RA patients and 38.7% of HC carry only one SE copy (OR=1.3; 95% CI=1.0-1.7; p=0.053), while the presence of two SE copies was detected in 19.9% of RA patients and 2.1% of HC (OR=11.7; 95% CI=6.4-21.4; p<0.001) (**Figure 1**, **Table 2**).

**Table 2:** *HLA-DRB1* genotype frequencies in a Chilean cohort of RA patients and HC.

Genotype	RA patients Number (frequency)	HC Number (frequency)
***Other/Other (X/X)***	**77 (21.0%)**	**162 (26.0%)**
***Other/”Shared Epitope” (X/SE)***	**112 (30.5%)**	**189 (30.3%)**
X/*01:01	15 (4.1%)	33 (5.3%)
X/*01:01·*01:02*	0 (0%)	2 (0.3%)
X/*01:02	9 (2.5%)	23 (3.7%)
X/*04:01	21 (5.7%)	13 (2.1%)
X/*04:04	15 (4.1%)	52 (8.4%)
X/*04:04·*04:08*	1 (0.3%)	0 (0%)
X/*04:05	16 (4.4%)	10 (1.6%)
X/*04:05·*04:08*	0 (0%)	1 (0.2%)
X/*04:05·*04:10*	0 (0%)	0 (0%)
X/*04:08	1 (0.3%)	0 (0%)
X/*04:10	0 (0%)	1 (0.2%)
X/*10:01	5 (1.4%)	8 (1.3%)
X/*14:02	29 (7.9%)	45 (7.2%)
X/*14:06	0 (0%)	0 (0%)
X/*14:13	0 (0%)	1 (0.2%)
***“Shared Epitope”/”Shared Epitope” (SE/SE)***	**73 (19.9%)**	**13 (2.1%)**
*01:01/*01:01	6 (1.6%)	0 (0%)
*01:01/*01:02	0 (0%)	1 (0.2%)
*01:01/*04:01	7 (1.9%)	0 (0%)
*01:01/*04:04·*04:08*	0 (0%)	1 (0.2%)
*01:01/*04:05	1 (0.3%)	0 (0%)
*01:01/*10:01	5 (1.4%)	0 (0%)
*01:01/*14:02	2 (0.5%)	2 (0.3%)
*01:02/*01:02	2 (0.5%)	0 (0%)
*01:02/*04:01	0 (0%)	3 (0.5%)
*01:02/*04:04	2 (0.5%)	2 (0.3%)
*01:02/*04:05	0 (0%)	1 (0.2%)
*01:02/*10:01	1 (0.3%)	0 (0%)
*01:02/*14:02	1 (0.3%)	0 (0%)
*04:01/*04:01	9 (2.5%)	0 (0%)
*04:01/*04:04	2 (0.5%)	1 (0.2%)
*04:01/*04:05	2 (0.5%)	0 (0%)
*04:01/*10:01	4 (1.1%)	0 (0%)
*04:01/*14:02	2 (0.5%)	0 (0%)
*04:04/*04:04	8 (2.2%)	0 (0%)
*04:04/*04:05	5 (1.4%)	0 (0%)
*04:04/*10:01	1 (0.3%)	0 (0%)
*04:04/*14:02	2 (0.5%)	0 (0%)
*04:04/*14:13	2 (0.5%)	0 (0%)
*04:04·04:08/*04:05·*04:10*	1 (0.3%)	0 (0%)
*04:05/*04:05	1 (0.3%)	0 (0%)
*04:05/*10:01	3 (0.8%)	0 (0%)
*04:08/*14:02	1 (0.3%)	0 (0%)
*10:01/*10:01	2 (0.5%)	2 (0.3%)
*10:01/*14:02	1 (0.3%)	0 (0%)
***Other/Protective (X/PR)***	**39 (10.6%)**	**183 (29.4%)**
X/*01:03	0 (0%)	1 (0.2%)
X/*04:02	4 (1.1%)	3 (0.5%)
X/*11:01	5 (1.4%)	65 (10.4)
X/*11:02	0 (0%)	3 (0.5%)
X/*11:04	1 (0.3%)	12 (1.9%)
X/*12:01	2 (0.5%)	3 (0.5%)
X/*13:01	7 (1.9%)	38 (6.1%)
X/*13:01·*13:02*	1 (0.3%)	2 (0.3%)
X/*13:02	1 (0.3%)	14 (2.3%)
X/*13:05	0 (0%)	0 (0%)
X/*16:01	0 (0%)	9 (1.4%)
X/*16:01·*16:02*	1 (0.3%)	1 (0.2%)
X/*16:02	17 (4.6%)	32 (5.1%)
***Protective/Protective (PR/PR)***	**13 (3.5%)**	**24 (3.9%)**
*04:02/*1301·*1302*	0 (0%)	1 (0.2%)
*11:01/*11:01	6 (1.6%)	5 (0.8%)
*11:01/*11:04	0 (0%)	1 (0.2%)
*11:01/*13:02	0 (0%)	4 (0.6%)
*11:01/*13:05	1 (0.3%)	0 (0%)
*11:01/*16:01	0 (0%)	2 (0.3%)
*11:01/*16:02	3 (0.8%)	2 (0.3%)
*11:02/*16:02	0 (0%)	1 (0.2%)
*13:01/*13:01	0 (0%)	3 (0.5%)
*13:01/*13:02	0 (0%)	1 (0.2%)
*13:01/*16:02	1 (0.3%)	2 (0.3%)
*13:02/*16:02	0 (0%)	1 (0.2%)
*13:05/*16:01	0 (0%)	1 (0.2%)
*13:05/*16:01·*16:02*	1 (0.3%)	0 (0%)
*16:02/*16:02	1 (0.3%)	0 (0%)
***“Shared Epitope”/Protective (SE/PR)***	**53 (14.4%)**	**52 (8.4%)**
*01:01/*04:02	0 (0%)	1 (0.2%)
*01:01/*11:01·*11:04*	0 (0%)	2 (0.3%)
*01:01/*11:04	0 (0%)	3 (0.5%)
*01:01/*13:01	2 (0.5%)	0 (0%)
*01:01/*13:05	2 (0.5%)	0 (0%)
*01:01/*16:02	2 (0.5%)	8 (1.3%)
*01:02/*11:01	0 (0%)	2 (0.3%)
*01:02/*11:04	0 (0%)	3 (0.5%)
*01:02/*13:01	0 (0%)	1 (0.2%)
*01:02/*16:02	1 (0.3%)	1 (0.2%)
*04:01/*11:01	0 (0%)	4 (0.6%)
*04:01/*13:01	1 (0.3%)	0 (0%)
*04:01/*13:02	3 (0.8%)	0 (0%)
*04:01/*16:02	8 (2.2%)	0 (0%)
*04:04/*04:02	2 (0.5%)	0 (0%)
*04:04/*11:01	2 (0.5%)	0 (0%)
*04:04/*13:02	9 (2.5%)	0 (0%)
*04:04/*16:02	1 (0.3%)	2 (0.3%)
*04:04·04:08/*11:01·*11:04*	0 (0%)	1 (0.2%)
*04:04·04:08/*11:18·*11:19*	1 (0.3%)	0 (0%)
*04:04·04:08/*13:01·*13:02*	1 (0.3%)	0 (0%)
*04:05/*01:03	2 (0.5%)	0 (0%)
*04:05/*11:01	2 (0.5%)	0 (0%)
*04:05/*12:01	1 (0.3%)	3 (0.5%)
*04:05/*13:02	0 (0%)	2 (0.3%)
*04:05/*16:02	1 (0.3%)	0 (0%)
*04:05·04:10/*13:01·*13:02*	2 (0.5%)	2 (0.3%)
*04:08/*11:01	2 (0.5%)	0 (0%)
*04:08/*16:02	1 (0.3%)	0 (0%)
*04:10/*13:01	0 (0%)	2 (0.3%)
*10:01/*11:01	1 (0.3%)	5 (0.8%)
*10:01/*11:04	0 (0%)	1 (0.2%)
*10:01/*13:01	1 (0.3%)	0 (0%)
*14:02/*04:02	0 (0%)	2 (0.3%)
*14:02/*11:01	3 (0.8%)	2 (0.3%)
*14:02/*11:04	0 (0%)	1 (0.2%)
*14:02/*13:01	0 (0%)	1 (0.2%)
*14:02/*13:02	2 (0.5%)	0 (0%)
*14:02/*13:05	0 (0%)	1 (0.2%)
*14:02/*16:02	0 (0%)	1 (0.2%)
*14:06/*16:02	0 (0%)	1 (0.2%)

Alleles and genotypes absent in both RA patients and HC are omitted; “X” denotes any allele not classified as a “Shared Epitope” (SE) or Protective (PR) allele, which are not shown in detail. In bold is shown the information for all pooled genotype combinations (X/X, X/SE, SE/SE, X/PR, PR/PR, and SE/PR). RA, rheumatoid arthritis; HC, healthy controls.

**Figure 2:**
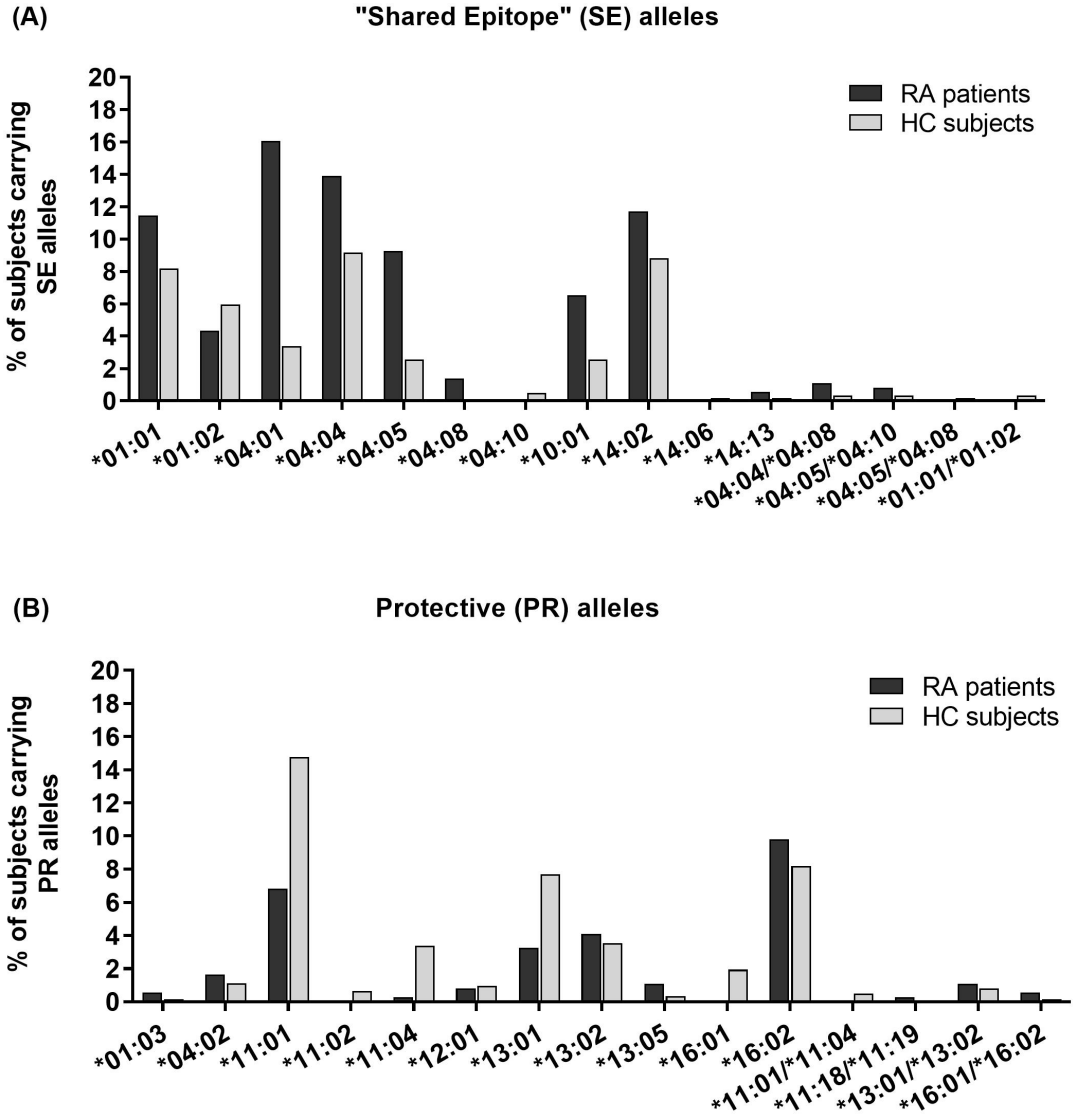
Percentage of rheumatoid arthritis (RA) patients and healthy controls (HC) carrying each **(A)** “Shared Epitope” (SE) or **(B)** Protective (PR) *HLA-DRB1* allele. The x-axis lists each allele, while the y-axis indicates the percentage of subjects carrying that allele. Black bars represent RA patients, and gray bars represent HC. Alleles not detected in either cohort are excluded.

Specific genotype frequencies in RA patients and HC are shown in **Table 2**. HLA-DR*04:01 was the most frequent SE allele among RA patients, followed by *04:04 and *14:02 (16.1%, 13.9% and 11.7% of all patients, respectively). Meanwhile, the most frequent SE allele in HC was *04:04, followed by *14:02 and *01:01 (9.2%, 8.8% and 8.2% of all individuals, respectively) (**Figure 2**, **Table 3**).

**Table 3:** Association between RA and *HLA-DRB1* genotypes carrying at least one copy of a “Shared Epitope” (SE) allele, protective (PR) allele, or non-SE/non-PR (X) allele, in Chilean individuals.

	*HLA-DRB1* allele	HLA-DR β1 amino acid sequence at positions 70-74	% RA patients	% healthy controls	OR	95% CI		p
**SE alleles**	*01:01	**Q R R A A**	11.5	8.2	1.5	0.9 -	2.2	0.090
	*01:02	**Q R R A A**	4.4	5.9	0.7	0.4 -	1.3	0.286
	*04:04	**Q R R A A**	13.9	9.2	1.6	1.1 -	2.4	0.021
	*04:05	**Q R R A A**	9.3	2.6	3.9	2.1 -	7.1	<0.001
	*04:08	**Q R R A A**	1.4	0	18.9	1.0 -	343.2	0.047
	*04:10	**Q R R A A**	0	0.5	1.0	1.0 -	1.0	0.299
	*14:02	**Q R R A A**	11.7	8.8	1.4	0.9 -	2.1	0.142
	*14:06	**Q R R A A**	0	0.2	1.0	1.0 -	1.0	1.000
	*14:13	**Q R R A A**	0.5	0.2	3.4	0.3 -	37.7	0.559
	*10:01	**R R R A A**	6.5	2.6	2.7	1.4 -	5.1	0.002
	*04:01	**Q K R A A**	16.1	3.4	5.5	3.3 -	9.2	<0.001
**PR alleles**	*11:01	D R R A A	6.8	14.8	0.4	0.3 -	0.7	<0.001
	*11:04	D R R A A	0.3	3.4	0.1	0 -	0.6	0.001
	*12:01	D R R A A	0.8	1.0	0.9	0.2 -	3.4	1.000
	*13:05	D R R A A	1.1	0.3	3.4	0.6 -	18.8	0.202
	*16:01	D R R A A	0	1.9	0.1	0 -	1.1	0.067
	*16:02	D R R A A	9.8	8.2	1.2	0.8 -	1.9	0.384
	*01:03	D E R A A	0.5	0.2	3.4	0.3 -	37.7	0.559
	*04:02	D E R A A	1.6	1.1	1.5	0.5 -	4.4	0.567
	*11:02	D E R A A	0	0.6	0.2	0 -	3.5	0.262
	*13:01	D E R A A	3.3	7.7	0.4	0.2 -	0.8	0.005
	*13:02	D E R A A	4.1	3.5	1.2	0.6 -	2.3	0.656
**X alleles**	*15:01	Q A R A A	9.5	14.1	0.6	0.4 -	1.0	0.035
	*15:02	Q A R A A	3.0	1.0	3.2	1.2 -	8.7	0.017
	*04:07	Q R R A E	9.5	19.9	0.4	0.3 -	0.6	<0.001
	*03:01	Q K R G R	12.3	18.6	0.6	0.4 -	0.9	0.009
	*07:01	D R R G Q	7.6	19.9	0.3	0.2 -	0.5	<0.001
	*08:02	D R R A L	5.5	13.3	0.4	0.2 -	0.6	<0.001
	*09:01	R R R A E	9.8	4.2	2.5	1.5 -	4.2	<0.001

Alleles showing a significant positive or negative association with RA are highlighted in dark or light gray, respectively. Underlined amino acid residues indicate differences from the SE sequence at positions 70-74. Residues shown in bold correspond to the SE motif at positions 70-74. Alleles not detected in either RA patients or HC are excluded. RA: rheumatoid arthritis; OR: odds ratio; CI: confidence interval.

The association between the disease and the presence of a specific *HLA-DRB1* allele was calculated considering the frequency of RA subjects positive and negative for at least one copy of the allele and the correlative data for HC. The SE alleles that showed a significant association with RA were: *04:01 (OR=5.5; 95% CI=3.3-9.2; p<0.001), *04:04 (OR=1.6; 95% CI=1.1-2.4; p=0.021), *04:05 (OR=3.9; 95% CI=2.1-7.1; p<0.001), *04:08 (OR=18.9; 95% CI=1.0-343.2; p=0.047), and *10:01 (OR=2.7; 95% CI=1.4-5.1; p=0.002) (**Table 3**). In addition, two non-SE alleles showed a significant association with the disease, *09:01 (OR=2.5; 95% CI=1.5-4.2; p<0.001) and *15:02 (OR=3.2; 95% CI=1.2-8.7; p=0.017) (**Table 3**).

### 3.3 Frequencies of PR alleles in Chilean RA patients and HC

The frequencies of *HLA-DRB1* alleles associated with protection against RA were also analyzed. The following alleles were considered PR alleles: HLA-DRB1*01:03, *04:02, *11:01, *11:02, *11:04, *12:01, *13:01, *13:02, *13:05, *16:01, and *16:02 (21). When it was impossible to discriminate between *13:01, *13:02, *16:01, and *16:02 alleles, carriers were nonetheless considered positive for PR alleles.

At least one copy of a PR allele was found in 28.6% of RA patients and 41.6% of HC (OR=0.6; 95% CI=0.4-0.7; p<0.001). The presence of one PR copy was detected in 25.1% of RA patients and 37.7% of HC (OR=0.6; 95% CI=0.4-0.7; p<0.001), while the frequency of two PR copies was 3.5% for RA patients and 3.9% for HC (OR=0.9; 95% CI=0.5-1.8; p=0.804) (**Figure 1**, **Table 2**).

HLA-DRB1*11:01 was the most frequent PR allele among HC, followed by *16:02 and *13:01 (15.2%, 8.2%, and 7.7% of all HC, respectively), while in RA patients the most frequent PR allele was *16:02, followed by *11:01 and *13:02 (9.8%, 6.8%, and 4.1% of all patients, respectively) (**Figure 2**, **Table 2**).

The PR alleles that showed a significative association with the absence of the disease (protective effect) were HLA-DRB1*11:01 (OR=0.4; 95% CI=0.3-0.7; p<0.001), *11:04 (OR=0.1; 95% CI=0-0.6; p=0.001), and *13:01 (OR=0.4; 95% CI=0.2-0.8; p=0.005) (**Table 3**). Notably, some alleles which do not code for the DRRAA or DERAA sequences also showed significant association with protection against RA: *15:01 (OR=0.6; 95% CI=0.4-1.0; p=0.035), *04:07 (OR=0.4; 95% CI=0.3-0.6; p<0.001), *03:01 (OR=0.6; 95% CI=0.4-0.9; p=0.009), *07:01 (OR=0.3; 95% CI=0.2-0.5; p<0.001), and *08:02 (OR=0.4; 95% CI=0.2-0.6; p<0.001) (**Table 3**).

### 3.4 Association between *HLA-DRB1* alleles and clinical parameters in Chilean RA patients

Because all patients in the RA-cohort-II were anti-CCP positive and only RF and DAS28 data from RA-cohort-I were available, only the latter was used to study the relationship between clinical parameters and the presence of *HLA-DRB1* alleles.

The association between circulating autoantibodies and the presence of a specific *HLA-DRB1* allele was calculated considering the frequency of seropositivity and seronegativity among RA subjects. No significant associations were found between anti-CCP (OR=1.8; 95% CI=0.6-5.5; p=0.31) or RF seropositivity (OR=0.5; 95% CI=0.2-1.6; p=0.235) and the presence of at least one copy of SE alleles, or for most SE alleles assessed individually (data not shown). However, the SE allele *14:02 showed a significant association with anti-CCP seropositivity among RA patients (OR=1.4; 95% CI=1.2-1.6; p=0.033; **Supplementary Table 1**). On the other hand, the SE allele *01:01 showed a significant association with RF seronegativity (OR=0.2; 95% CI=0.1-0.8; p=0.024), while the non-SE allele *13:03 was associated with anti-CCP seronegativity (OR=0.1; 95% CI=0-0.6; p=0.017; **Supplementary Table 1**).

The presence of at least one copy of PR alleles also showed no association with anti-CCP (OR=0.9; 95% CI=0.3-2.8; p=0.767) or RF (OR=2.5; 95% CI=0.7-9.5; p=0.156) seropositivity among RA patients.

Finally, no associations were found between DAS28 values and the presence of at least one copy of SE (p=0.595) or PR (p=0.838) alleles in the analyzed RA cohort. However, we observed that low DAS28 values were significantly associated with the presence of the SE allele *04:04 (mean value of 2.8 versus 4.1 for *04:04 positive and negative RA patients, respectively; p=0.028; **Supplementary Table 2**).

## 4 Discussion

Although RA is a multifactorial disease, the presence of *HLA-DRB1* alleles encoding the SE remains the most important genetic risk factor (21). To date, a detailed study of the distribution of *HLA-DRB1* alleles in Chilean RA patients has not been addressed. This is crucial information to better understand the genetic landscape of RA in the country and the region, and the pathogenic mechanisms underlying disease development in this ethnically mixed population.

In the present study, we observed a higher frequency of SE alleles among Chilean RA patients (64.8%) than HC (40.8%). The proportion of RA patients carrying at least one SE allele was higher than that reported in Asian and African populations (<50%) (10, 33, 34) but lower than in Caucasian patients (>80%) (35, 36), aligning more closely with data from North and South American cohorts (50-75%) (14, 37, 38). We also found that when the SE is expressed in a double dose, the risk of developing the disease is higher, as previously described (10). In our examination of the Chilean population, *04:01 was the most frequent SE allele among RA patients, followed by *04:04 and *14:02, which is in agreement with prior reports of high *04:01 and *04:04 frequencies (25, 26). We also noted significant associations between RA and alleles *04:01, *04:04, *04:05, *04:08, and *10:01. Except for *04:05, these findings align with previous studies in Chilean populations (25, 26). The non-SE allele *09:01 was also linked to RA in our cohort, consistent with studies in Chilean (25) and Asian populations (39). This allele has also been implicated in other autoimmune diseases such as systemic lupus erythematosus (39) and type 1 diabetes (40). Although *09:01 differs from *10:01 by a single amino acid substitution (alanine for glutamic acid at position 74), there is no conclusive evidence that the RRRA sequence at positions 70–73 alone confers susceptibility to RA. However, *09:01 is in linkage disequilibrium with *HLA-DRB4*, a paralog of *HLA-DRB1* that also encodes for an HLA-DRβ chain. *HLA-DRB4*, found exclusively in DR4, DR7, and DR9 haplotypes, has been associated with a more aggressive RA progression (41). Our cohort also revealed an association between *15:02 and RA. Although DRB1*15 alleles have traditionally been considered as low risk alleles (42), they have been linked to pulmonary complications in RA (43, 44). Future studies with comprehensive records of extra-articular manifestations of the disease would help to clarify this association.

Interestingly, the *14:02 allele emerged as over-represented in our cohorts, ranking as the third most frequent allele in RA patients and the second in HC. Notably, the *14:02 allele has been previously associated with RA in various American populations, including Native Americans (16), Mexicans (14), and Peruvians (15). A study in Indigenous North Americans showed that the *14:02 allele confers substantial risk for RA due to a combination of serine residues at positions 11 and 13, which would allow HLA-DR to accommodate both citrulline and arginine at the P4 pocket, potentially enhancing anti-citrullinated antigen T cell responses and ACPA development (45). In line with these observations, we found that the *14:02 allele was significantly associated with the presence of anti-CCP autoantibodies.

Although many studies attribute the strong association between HLA and anti-CCP RA disease to the presence of the SE in the HLA-DR molecule, other pieces of evidence suggest that the SE hypothesis alone would not fully explain *HLA-DRB1* associations with the disease. Indeed, five amino acid positions across three HLA genes (positions 11, 71, and 74 in *HLA-DRB1*, as well as position 9 in *HLA-B* and *HLA-DPB1*) have been shown to collectively explain most of the HLA region’s contribution to seropositive RA in Europeans (8). Conditional haplotype analyses demonstrated that B*08/Asp-9 and/or *HLA-DPB1*/Phe-9 alleles increase the RA risk on specific *HLA-DRB1* backgrounds such as *09:01 and *15:02, with position 11 in *HLA-DRB1* being determinant to this effect: while Val-11 and Leu-11 confer high risk, Ser-11 is highly protective for the disease. This is particularly interesting in light of our dataset, where SE alleles *04:04, *04:05, *04:08, *10:01, and *04:01 (all containing Val or Leu at position 11) were associated with RA risk, while *03:01, *08:02, *11:01, *11:04, and *13:01 (all containing Ser-11) showed protective effects. Studies focusing on Asian and European populations with ACPA-positive RA have also linked His-13 to increased RA risk, which is reinforced by our results regarding *04:04, *04:05, and *04:01 alleles (12). The risk effect of Val-11 and His-13, and the protective effect of Ser-11, have been confirmed in autoantibody-positive African RA patients as well (46). In Japanese patients with early RA, *04:05 (encoding Val at position 11) correlated with higher disease risk and anti-CCP seropositivity, whereas *09:01 (encoding Asp at position 11) appeared more commonly in anti-CCP-negative patients, underscoring how position 11 variants can differentially influence seropositivity (34).

On the protective side, PR alleles were more frequent in HC (41.6%) than in RA patients (28.6%), and their presence was negatively correlated with the disease, confirming previous reports (19). Specifically, *11:01, *11:04, and *13:01 alleles were negatively associated with RA, which is consistent with observations in Argentinian (47) and European ACPA-positive cohorts (48). Interestingly, additional alleles not classically categorized as protective (*15:01, *04:07, *03:01, *07:01, and *08:02) also showed negative associations with RA. Previous reports in Caucasian RA populations have attributed the protective role of *07:01 and *15:01 to the presence of an isoleucine at position 67 of the HVR3, which is also present in PR alleles *01:03, *04:02, *11:02, *13:01, *13:02, and *12:01 (49). Our results for *07:01 and *08:02 alleles are consistent with previous evidence showing a negative association with ACPA-positive RA in Latin American admixed populations (25). Although *04:07 has been considered neutral for RA (49), our findings suggest a protective effect, possibly linked to a negatively charged glutamic acid at position 74 of the HVR3, similar to that at position 71 in *04:02, and contrasting with the positively charged residues in SE alleles (50). DRB1*03:01 has been associated with ACPA-negative RA but exhibits a protective effect for ACPA-positive RA (51), paralleling our data (where 54% of the RA cohort was ACPA-positive). These findings imply that HLA-DR-mediated protection against RA may not always depend on DRRAA or DERAA sequences at P4. Interestingly, although we did not find a correlation between PR alleles with seronegativity, as the literature suggests for presence of HLA-DR3 or DERAA-encoding alleles (52), we found a significant association between *13:03 (a non-PR allele) and the absence of anti-CCP autoantibodies. Another study examining ACPA-positive versus ACPA-negative RA in European-descent cohorts revealed that Ser-11 and Leu-11 in *HLA-DRB1* and Asp9 in *HLA-B* can drive risk for ACPA-negative RA (51), potentially explaining our findings for *13:03 which also contains Ser-11. Indeed, HLA-DRB1*13 alleles have been noted to affect the ACPA status, conferring protection for ACPA-positive RA and, in combination with HLA-DRB1*03, reducing the risk of ACPA-negative RA (53). It should be noted that only part of our RA cohort was used to explore associations with autoantibody profiles, as RA-cohort-II was entirely ACPA-positive. Consequently, the associations observed in this study, particularly for seronegative patients, should be interpreted with caution and verified in larger cohorts.

Studying the role of HLA alleles in driving autoantibody production and influencing disease outcomes in RA is not easy. Actually, distinct HLA associations for RA subsets have also been explored by specific autoantibody profiles, where Asp-9 in *HLA-B* has emerged as a shared risk factor for both ACPA-positive and ACPA-negative RA but with variations according to the presence of different autoantibody clusters (54). In this context, HLA-DRB1*03:01, which was revealed to be protective in our cohort, has been identified as a primary driver of *HLA-DRB1* associations in second-generation anti-cyclic citrullinated peptide (CCP2) negative RA patients, but not in CCP2-positive RA patients, demonstrating similar patterns to *HLA-B*/Asp-9 (51, 54). This illustrates how specific alleles and particular amino acid residues may define diverse autoantibody profiles, further refining the categorization of RA beyond simple seropositivity and seronegativity. Nevertheless, it is widely accepted that the evolving autoimmune response in RA is closely linked to the presence of SE-expressing HLA-DR molecules, which are distinguished as exhibiting a high affinity for citrullinated peptides. Within RA-inflamed joints, citrullinated peptides can be recognized as foreign and preferentially presented by SE-expressing HLA-DR to autoreactive CD4+ T cells (55–58). Subsequently, these activated CD4+ T cells could facilitate the activation of autoreactive B cells, culminating in the production and secretion of autoantibodies, the hallmark of RA (59–62).

Although most of the *HLA-DRB1* effect on disease severity is mediated by ACPA, recent evidence has shown that specific *HLA-DRB1* amino acids can also modulate inflammatory markers (like C-reactive protein) and clinical parameters (e.g., DAS28 or the Swollen Joint Count) through different pathways, some of which are likely to be independent of ACPA (5, 63). For example, large-scale research in Japanese RA patients found that DRB1*04:05 is significantly associated with radiographic damage (Sharp/van der Heijde score) in anti-CCP-positive RA, even after adjusting for the DAS28, implying subclinical mechanisms not fully captured by conventional disease activity metrics (64, 65). In a separate Caucasian RA cohort, haplotypes containing HLA-DRB1*04:04 and HLA-DRB1*01:01, along with six single-nucleotide polymorphisms in the lymphotoxin alpha and tumor necrosis factor DNA-coding region, correlated strongly with therapeutic response in early RA (66). Although in our dataset HLA-DRB1*04:04 appears to be associated with lower DAS28 values, which is at odds with the literature, this should also be taken with caution given the small sample size of patients with available DAS-28 data in our cohort.

Despite the strengths of our study, several limitations should be acknowledged. First, the HC were selected from a general population cohort that was not screened for autoimmune laboratory tests, potentially allowing for subclinical autoimmune conditions to go undetected. Second, the PCR-SSO method only provided intermediate-resolution typing of *HLA-DRB1* alleles, meaning that the reported associations could not be confirmed at the highest possible resolution; however, according to comparative studies, PCR-SSO and next generation sequencing (NSG) methodologies have an acceptable concordance for *HLA-DRB1* alleles (67, 68). Third, risk-factor information, including tobacco index and biomass-burning smoke exposure, were unavailable for some participants, thereby restricting the scope of our analyses, in particular given the well-described interaction between tobacco smoking, SE alleles and ACPA in RA (60). A further limitation related to our autoantibody analyses was the lack of quantitative data for anti-CCP and RF antibodies, which prevented us from exploring potential associations between antibody levels and specific *HLA-DRB1* alleles. Finally, the cross-sectional design prevented us from performing longitudinal analysis between genetic factors and disease progression. Future investigations incorporating more thoroughly screened populations, high-resolution genotyping approaches, expanded clinical and risk-factor data collection, and longitudinal follow-up will be essential to address these issues.

Considering the clear variations in allele frequencies among different ethnic groups, ongoing research is essential to refine the understanding of how genetics may influence RA establishment and evolution. In this context, our findings provide updated data on *HLA-DRB1* distributions in the Chilean population, information that is not only valuable for understanding the disease immunopathogenesis but also holds promise for the development of personalized approaches to RA prevention and treatment.

## Data Availability

The original contributions presented in the study are included in the article/**Supplementary Material**. Further inquiries can be directed to the corresponding authors.
