# Supplementary material for: HLA-DRB1 allele distribution in Chilean population: insights into rheumatoid arthritis susceptibility and protection
Source: Front Immunol. 2025 May 13;16:1594723. doi: 10.3389/fimmu.2025.1594723 (PMC12106328; doi:10.3389/fimmu.2025.1594723)
Supplement: Supplementary file 1 [file Table1.docx]

**Supplementary Table 1.** Association between ACPA seropositivity or seronegativity and *HLA-DRB1* genotypes carrying at least one copy of a “Shared Epitope” (SE) allele, a protective (PR) allele, or a non-SE/non-PR (X) allele in Chilean RA patients.

|  | ***HLA-DRB1*  allele** | **% of ACPA+**  **RA patients** | **% of ACPA-**  **RA patients** | **OR** | **95% CI** | | **p-value** |
| --- | --- | --- | --- | --- | --- | --- | --- |
| **SE alleles** | **01:01* | 8.6 | 13.3 | 0.6 | 0.1 - | 3.4 | 0.626 |
|  | **01:02* | 7.1 | 13.3 | 0.5 | 0.1 - | 2.9 | 0.602 |
|  | **04:04* | 8.6 | 6.7 | 1.3 | 0.2 - | 11.8 | 1 |
|  | **04:05* | 11.4 | 13.3 | 0.8 | 0.2 - | 4.4 | 1 |
|  | **04:08* | 1.4 | 0 | 1.0 | 1.0 - | 1.0 | 1 |
|  | **14:02* | 25.7 | 0 | 1.4 | 1.2 - | 1.6 | 0.033 |
|  | **10:01* | 4.3 | 6.7 | 0.6 | 0.1 - | 6.5 | 0.547 |
|  | **04:01* | 8.6 | 6.7 | 1.3 | 0.2 - | 11.8 | 1 |
| **PR alleles** | **11:01* | 5.7 | 6.7 | 0.9 | 0.1 - | 8.2 | 1 |
|  | **11:04* | 1.4 | 0 | 1.0 | 1.0 - | 1.0 | 1 |
|  | **12:01* | 1.4 | 6.7 | 0.2 | 0 - | 3.4 | 0.324 |
|  | **16:02* | 10.0 | 6.7 | 1.6 | 0.2 - | 13.7 | 1 |
|  | **01:03* | 1.4 | 0 | 1.0 | 1.0 - | 1.0 | 1 |
|  | **04:02* | 2.9 | 13.3 | 0.2 | 0 - | 1.5 | 0.142 |
|  | **13:02* | 4.3 | 0 | 1.0 | 1.0 - | 1.1 | 1 |
| **X alleles** | **15:01* | 11.4 | 13.3 | 0.8 | 0.2 - | 4.4 | 1 |
|  | **15:02* | 1.4 | 0 | 1.0 | 1.0 - | 1.0 | 1 |
|  | **04:07* | 12.9 | 26.7 | 0.4 | 0.1 - | 1.6 | 0.232 |
|  | **03:01* | 11.4 | 6.7 | 1.8 | 0.2 - | 15.6 | 1 |
|  | **07:01* | 11.4 | 0 | 1.1 | 1.0 - | 1.2 | 0.340 |
|  | **08:02* | 14.3 | 20.0 | 0.7 | 0.2 - | 2.8 | 0.692 |
|  | **13:03* | 4.3 | 26.7 | 0.1 | 0 - | 0.6 | 0.017 |

*Alleles showing a significant association with ACPA seropositivity (dark gray) or seronegativity (light gray) are highlighted. RA: rheumatoid arthritis; ACPA: anti-citrullinated peptide/protein antibodies. OR: odds ratio; CI: confidence interval.*
