# Supplementary material for: HLA-DRB1 allele distribution in Chilean population: insights into rheumatoid arthritis susceptibility and protection
Source: Front Immunol. 2025 May 13;16:1594723. doi: 10.3389/fimmu.2025.1594723 (PMC12106328; doi:10.3389/fimmu.2025.1594723)
Supplement: Supplementary file 2 [file Table2.docx]

**Supplementary Table 2.** Association between DAS28 disease activity scores and *HLA-DRB1* genotypes carrying at least one copy of a “Shared Epitope” (SE) allele, a protective (PR) allele, or a non-SE/non-PR (X) allele in Chilean RA patients.

|  | ***HLA-DRB1*  allele** | **DAS28 mean value in allele negative RA patients** | **DAS28 mean value in allele positive RA patients** | **p-value** |
| --- | --- | --- | --- | --- |
| **SE alleles** | **01:01* | 3.9 | 4.4 | 0.250 |
|  | **01:02* | 4.0 | 4.2 | 0.684 |
|  | **04:04* | 4.1 | 2.8 | 0.028 |
|  | **04:05* | 4.0 | 4.2 | 0.692 |
|  | **04:08* | 4.0 | 4.7 | 0.647 |
|  | **14:02* | 4.1 | 3.7 | 0.327 |
|  | **10:01* | 4.0 | 4.7 | 0.303 |
|  | **04:01* | 4.0 | 3.7 | 0.515 |
| **PR alleles** | **11:01* | 4.0 | 4.5 | 0.347 |
|  | **12:01* | 4.0 | 3.8 | 0.807 |
|  | **13:05* | 4.0 | 3.3 | 0.637 |
|  | **16:02* | 4.0 | 3.8 | 0.745 |
|  | **01:03* | 4.0 | 5.9 | 0.083 |
|  | **04:02* | 4.0 | 2.7 | 0.142 |
|  | **13:02* | 4.0 | 3.0 | 0.287 |
| **X alleles** | **15:01* | 4.0 | 3.8 | 0.594 |
|  | **15:02* | 4.0 | 6.1 | 0.180 |
|  | **04:07* | 4.0 | 4.0 | 0.935 |
|  | **03:01* | 4.0 | 4.0 | 0.959 |
|  | **07:01* | 4.1 | 3.2 | 0.081 |
|  | **08:02* | 4.0 | 4.1 | 0.818 |
|  | **13:03* | 4.0 | 4.2 | 0.702 |

*Alleles showing a significant association with DAS28 mean values are highlighted in light gray. RA: rheumatoid arthritis; DAS28: Disease Activity Score based on 28 joint counts.*
